# Effects of Elevational Gradient on Biomass Allocation Patterns of Moso Bamboo Forests in Central-Southern Jiangxi, China

**DOI:** 10.3390/plants15142190

**Published:** 2026-07-17

**Authors:** Shan Li, Jialin Fan, Xiaotong Liu, Jiajun Liu, Zhoubin Huang, Jingyao Zhang, Guanglu Liu

**Affiliations:** 1 International Centre for Bamboo and Rattan/Key Laboratory of National Forestry and Grassland Administration for Bamboo & Rattan Science and Technology, Beijing 100102, China; 13041074829@163.com (S.L.); xiaotongliu@icbr.ac.cn (X.L.); 18713425485@163.com (J.L.); huangzhoubin@icbr.ac.cn (Z.H.); 15227393159@163.com (J.Z.); 2 Beijing Jingnong Holding Group Co., Ltd., Beijing 100070, China; f1622863635@163.com

**Keywords:** Moso bamboo (*Phyllostachys edulis*), elevational gradient, biomass allocation, diameter at breast height (DBH), stand structure, allometric relationship, decoupling mechanism

## Abstract

This study aimed to investigate the accumulation of aboveground biomass, organ allocation patterns, and their driving mechanisms in Moso bamboo (*Phyllostachys edulis*) forests along different elevational gradients and to compare regional differences in growth processes. A total of 54 sample plots were established along an elevational gradient from 50 to 550 m across three different regions, with 100 m elevational intervals. Two-way ANOVA, regression analysis, Tukey’s HSD multiple comparisons, and generalized additive models (GAMs) were used to examine distribution patterns. (1) Individual bamboo biomass followed a unimodal pattern with increasing elevation, peaking at 150 m (16.20 ± 3.88 kg culm^−1^), which was significantly higher than at other elevations (*p* = 0.048). Allometric covariance analysis showed that the *b* value did not differ significantly among elevations (*p* = 0.882), indicating a stable diameter at breast height (DBH)-biomass relationship. (2) Stand biomass was highest at 50 m (34.75 ± 10.97 t·ha^−1^) and declined with elevation to 18.54 ± 7.13 t·ha^−1^ at 550 m, revealing a decoupling from the elevational trend of individual biomass. (3) Branch and leaf dry mass allocation exhibited a “higher at both ends, lower in the middle” pattern. Culm allocation was highest at 150 m (80.3%), though differences among elevations were not statistically significant (*p* = 0.591). (4) Stand density decreased with elevation, while mean DBH first increased and then decreased, reaching a maximum at 450 m (9.77 cm). Differences in stand density and DBH among elevations were highly significant (*p* < 0.001). (5) ANCOVA showed that after controlling for mean DBH, the effect of elevation on individual biomass was substantially weakened (*p* = 0.051), with partial η^2^ declining from 0.48 to 0.21 (a 56% reduction), indicating that DBH accounted for a substantial portion of the elevation effect on individual biomass.The individual biomass of Moso bamboo in central-southern Jiangxi peaked at approximately 150 m elevation. Elevation was associated with biomass mainly through its association with DBH (a size effect), rather than through changes in allocation ratios or allometric relationships. The pathway “elevation→DBH→individual biomass” appears to be the primary mediating pathway explaining the decoupling, although a causal interpretation requires further experimental validation. These findings provide a theoretical basis for elevation-differentiated management of Moso bamboo forests.

## 1. Introduction

Moso bamboo (*Phyllostachys edulis*) is an important forest resource in southern China. Owing to its rapid growth, early maturity, and wide range of uses, it plays an irreplaceable role in timber production, bamboo shoot production, and ecological protection [1,2]. According to the 2024 China Natural Resources Bulletin, the total area of bamboo forests in China is 6.9779 million ha, of which Moso bamboo forests account for approximately 70%. The annual output value of China’s bamboo industry exceeds CNY 300 billion, making it a pillar industry for increasing farmers’ income and promoting rural revitalization in mountainous areas of southern China [1]. As the most important raw material source for the bamboo industry, Moso bamboo is of great economic significance. However, the productivity of Moso bamboo forests exhibits pronounced spatial heterogeneity. Even in adjacent areas, biomass per unit area and bamboo yield often differ by several-fold, which constrains precision management, quality improvement, and efficiency enhancement [3].

Elevational gradient is a key environmental factor driving variation in plant productivity and community structure in mountain ecosystems [4], and it is also an important driver of productivity heterogeneity in Moso bamboo forests. With increasing elevation, air temperature decreases, the growing season shortens, solar radiation intensifies, and wind speed increases, leading to adaptive adjustments in plant photosynthetic physiology, resource acquisition strategies, and biomass allocation patterns [5]. The mid-domain effect, a geometric null model originally proposed to explain species richness patterns along bounded spatial domains, predicts that species richness or individual abundance peaks at the center of a bounded domain due to geometric constraints alone [6,7]. Recent studies have extended this concept to plant biomass, showing that non-native plants may exhibit hump-shaped biomass patterns along elevation gradients partly due to spatial mid-domain effects. However, whether such geometric constraints contribute to the biomass patterns of native clonal plants like Moso bamboo remains unknown. Previous studies have shown that aboveground biomass of tree species generally declines with increasing elevation, although some species exhibit productivity peaks at mid-elevations [8,9]. Biomass allocation patterns reflect the core strategies by which plants adapt to environmental change [10]. According to the optimal allocation hypothesis, plants tend to preferentially allocate biomass to organs responsible for acquiring the most limiting resources [11]. Under high-elevation conditions, low temperature may constrain photosynthetic enzyme activity and nutrient uptake, prompting plants to increase investment in leaves to maintain carbon acquisition capacity. At the same time, increased risks associated with wind load and snow pressure may reduce investment in tall supporting structures [12]. On the other hand, stand structure, including mean diameter at breast height (DBH), density, and age composition, represents an intermediate link between environmental gradients and stand productivity [13]. Elevation may indirectly regulate stand biomass by altering individual bamboo size (DBH) and population abundance (density). However, most existing studies remain at the level of correlation analysis and lack quantitative decomposition of structural driving effects [14,15].

Allometric theory provides an effective framework for analyzing the relationship between plant size and form [16,17]. The conservation or variation of the DBH–biomass allometric exponent can reflect plant “body size” strategies in response to environmental conditions [18]. As a clonal plant, Moso bamboo shares resources and disperses risk through its underground rhizome system, and its response to elevational heterogeneity may therefore differ from that of non-clonal plants [19]. Its physiological integration may confer a certain degree of conservatism in aboveground allocation strategies, but whether this characteristic is maintained across elevational gradients remains to be empirically tested. At present, studies on the effects of elevation on biomass distribution patterns in Moso bamboo remain relatively limited. Existing reports have mainly focused on single regions or discontinuous elevational sampling [20] and have primarily examined variation in total biomass, with insufficient attention to allocation patterns (trade-offs between branches, leaves, and culms), the mediating effects of stand structure (roles of DBH and density), and dynamic growth processes [21,22,23]. In addition, few comparative studies across multiple regions have explored how regional background conditions, such as soil parent material and management intensity, influence the elevational responses of Moso bamboo forests [24]. Based on the existing literature, we formulated the following hypotheses:

H1 individual bamboo biomass exhibits a unimodal (hump-shaped) pattern along the elevational gradient, with a peak at an intermediate elevation;

H2 elevation influences individual biomass primarily through changes in DBH (a size effect) rather than through shifts in biomass allocation ratios or allometric relationships;

H3 biomass allocation among organs (branches, leaves, and culms) remains relatively stable across elevations due to the clonal growth form of Moso bamboo.

In this study, Wan’an County, Jiangxi Province, was selected as the study area. Across three representative regions, six continuous elevational gradients ranging from 50 to 550 m were established simultaneously, and 54 standard plots (20 m × 20 m) were set up. Organ biomass was determined using the whole-harvest method, and stand structural data were obtained through individual bamboo measurements. Two-way ANOVA, analysis of covariance, allometric equations, and generalized additive models (GAMs) were used to analyze the variation in the individual and stand-level biomass of Moso bamboo along the elevational gradient and to determine whether an optimal elevation exists. We further aimed to reveal the response patterns of biomass allocation among branches, leaves, and culms to elevational gradients; quantify the mediating roles of stand structure (DBH, density, and age composition) in the elevational variation of biomass; and elucidate regional differences in the DBH-age growth process of Moso bamboo. The results of this study may provide a theoretical basis for elevation-specific management of Moso bamboo forests and the cultivation of large-diameter bamboo.

## 2. Materials and Methods

### 2.1. Study Area

The study was conducted in Wan’an County, Ji’an City, Jiangxi Province, China (114°30′–115°5′ E, 26°8′–26°43′ N), located in the central-southern part of Jiangxi Province along the middle reaches of the Ganjiang River. The region belongs to the mid-subtropical humid monsoon climate zone and is characterized by a mild climate, abundant rainfall, sufficient sunshine, and a long frost-free period. The mean annual temperature is 18.5 °C, with an extreme maximum temperature of 40.2 °C and an extreme minimum temperature of −5.5 °C. Mean annual precipitation is 1380 mm, most of which occurs from April to June. Annual sunshine duration averages 1750 h, and the frost-free period is approximately 288 d. The terrain is dominated by low mountains and hills, with elevations mainly ranging from 100 to 800 m and a general topographic pattern of higher elevations in the south and lower elevations in the north. The main soil types are red soil and yellow-red soil, developed primarily from granite and sandstone-shale parent materials, with soil depths ranging from 40 to 80 cm and pH values of 4.5–6.0.

Wan’an County is one of the key production areas of Moso bamboo in Jiangxi Province, with an existing Moso bamboo forest area of 24,300 ha (365,000 mu) and a total standing culm number of 61.49 million. Moso bamboo resources are highly concentrated, with 76.2% distributed in Wufeng, Shaping, Gaobei, and Jiantou towns surrounding the county seat. These areas are all characterized by low mountain and hilly landforms with elevations ranging from 50 to 800 m, making them ideal for research on elevational gradients in Moso bamboo forests. Based on differences in the distribution of Moso bamboo resources and topographic characteristics, three representative regions were selected from these four towns for this study: Wufeng Town as Region A, Gaobei Town as Region B, and Jiantou Town as Region C (Table 1). The regions differ to some extent in soil parent material, soil depth, slope, and management intensity, thereby providing a natural gradient for analyzing the effects of regional background conditions on biomass patterns in Moso bamboo forests.

### 2.2. Plot Establishment

Within each region, six elevational classes were established along the elevational gradient: 50, 150, 250, 350, 450, and 550 m. At each elevational class, one representative study site was selected, and within each site, three 20 m × 20 m standard plots were established as replicates, yielding a total of 54 plots. During plot selection, forest edges, steep slopes, landslide areas, and sites with obvious human disturbance were avoided to ensure that all stands were pure Moso bamboo forests with intact stand conditions and good representativeness. The four corners of each plot were permanently marked with PVC pipes, and information on geographic coordinates, elevation, slope, aspect, soil type, and soil depth was recorded. During plot selection, we prioritized stands with no recorded major disturbances (thinning, fertilization, or clearcutting) in the preceding five years, based on interviews with local forest managers and landowners. However, detailed quantitative records of past management practices were not available for all plots, and this represents a limitation of this study.

It should be noted that the nested design adopted in this study, in which three replicate plots were established within each study site, is a commonly used approach in field studies conducted in topographically complex areas to balance repeatability and operational feasibility. However, this design may lead to non-independence among the three plots within the same site because of their spatial proximity, potentially resulting in overestimation of the significance of some statistical parameters. To account for this potential effect, linear mixed-effects models (LMMs) were used for validation analysis, with “study site” included as a random effect in the model.

### 2.3. Individual Bamboo Measurements and Stand Structural Variables

All Moso bamboo culms within each plot were measured individually. Diameter at breast height (DBH; measured at 1.3 m, cm) was recorded, and culm age was determined. Age classes were classified according to culm color, sheath ring scars, and related morphological characteristics into Degree I (1-year-old), Degree II (2–3 years old), Degree III (4–5 years old), and Degree IV or above (≥6 years old). Mean DBH, coefficient of variation in DBH (CV = standard deviation/mean × 100%), stand density (culms ha^−1^), and the proportion of each age class were calculated for each plot.

### 2.4. Biomass Determination

Within each 20 m × 20 m plot, all living Moso bamboo culms with DBH > 4 cm were measured for DBH to establish the diameter-class frequency distribution (2 cm class intervals: 4–6, 6–8, 8–10, 10–12, 12–14, and >14 cm). Standard culms were then selected proportionally to the diameter-class distribution of each plot, ensuring that the size distribution of sampled culms matched that of the stand. In plots with more than five diameter classes, additional culms were selected to maintain representativeness. In total, 54 standard culms were harvested across all elevations. A Kolmogorov–Smirnov test confirmed that the DBH distribution of the sampled culms did not differ significantly from that of the standing culms in each plot (all *p* > 0.05), confirming their representativeness. Each standard culm was felled at ground level, and the fresh mass of each organ (branches, leaves, and culm) was weighed in the field. Samples of each organ were then taken to the laboratory and oven-dried at 85 °C to a constant weight to calculate dry-to-fresh mass ratios. Total plot biomass was estimated proportionally from the dry mass of each organ of the standard culms and the total number of culms in each plot. The formulas used were as follows:

(1) Mean diameter at breast height (DBH)

Mean DBH refers to the arithmetic mean of the DBH values of all Moso bamboo individuals with DBH > 4 cm within a plot and is used to characterize the average DBH level of the stand.

(1) $$\mathrm{DBH}=\frac{1}{n}\sum_{i=1}^{n}DBH_{i}$$

where DBH is the mean plot DBH (cm), DBH_i_ is the DBH of the ith bamboo culm (cm), and *n* is the total number of bamboo culms with DBH > 4 cm in the plot.

(2) Dry-to-fresh mass ratio

(2) $$\mathrm{Dry}\text{-}\mathrm{to}\text{-}\mathrm{fresh}\;\mathrm{mass}\;\mathrm{ratio}\;\mathrm{of}\;\mathrm{branches}\;\mathrm{and}\;\mathrm{leaves}=\frac{\mathrm{dry}\;\mathrm{mass}\;\mathrm{of}\;\mathrm{branches}+\mathrm{dry}\;\mathrm{mass}\;\mathrm{of}\;\mathrm{leaves}}{\mathrm{fresh}\;\mathrm{mass}\;\mathrm{of}\;\mathrm{branches}+\mathrm{fresh}\;\mathrm{mass}\;\mathrm{of}\;\mathrm{leaves}}$$

(3) $$Dry\text{-}to\text{-}fresh\;mass\;ratio\;of\;culm=\frac{dry\;mass\;of\;culm}{fresh\;mass\;of\;culm}$$

(3) Dry mass proportion

(4) $$Dry\;mass\;proportion\;of\;branches\;and\;leaves=\frac{total\;dry\;mass\;of\;branches\;and\;leaves}{total\;aboveground\;dry\;mass}\times 100\%$$

(5) $$Dry\;mass\;proportion\;of\;culm=\frac{dry\;mass\;of\;culm}{total\;aboveground\;dry\;mass}\times 100\%$$

(4) Individual biomass (MB)

The aboveground biomass of an individual standard bamboo culm was calculated as the sum of the dry mass of all organs:

(6) $$\mathrm{MB}\;={\;W}_{branch}\;+\;W_{leaf}\;+\;W_{culm\;}$$

where MB is the aboveground biomass of an individual standard bamboo culm (kg), W_branch_ is the oven-dried mass of branches (kg), W_leaf_ is the oven-dried mass of leaves (kg), and W_culm_ is the oven-dried mass of the culm (kg).

(5) Stand aboveground biomass (AGB)

Stand aboveground biomass refers to the total dry mass of aboveground components (culms, branches, and leaves) per unit area (1 ha) in a Moso bamboo forest and is expressed in t·ha^−1^. It was estimated based on the individual biomass of the standard bamboo culms and stand density:

(7) $$\mathrm{AGB}\;=\;\frac{\mathrm{MB}\;\times \;n}{40}$$

where AGB is the stand aboveground biomass (t·ha^−1^), MB is the aboveground biomass of an individual standard bamboo culm (kg), and n is the total number of bamboo culms in the plot. In Equation (7), the divisor 40 converts plot-level biomass (kg per 400 m^2^) to stand-level biomass (t·ha^−1^). Each plot is 20 m × 20 m = 400 m^2^ = 0.04 ha. Since *MB* is in kg and *n* is the number of culms per plot, the stand biomass in t·ha^−1^ is calculated as (*MB* × *n*)/(0.04 × 1000) = (*MB* × *n*)/40.

### 2.5. Data Analysis

Microsoft Excel 2019 (Microsoft Corporation, Redmond, WA, USA) and R 4.3.1 (R Core Team, Vienna, Austria) were used for data processing and statistical analysis. Two-way analysis of variance was performed to test the effects of region, elevation, and their interaction on each variable, and post hoc multiple comparisons were conducted using Tukey’s honestly significant difference (HSD) test (α = 0.05) to control the family-wise error rate. Because the nested plot design may introduce spatial pseudoreplication, linear mixed-effects models (LMMs; model form: response variable~elevation + region + (1|site)) were used for validation, and conservative analyses were also performed using site means (*n* = 3 for each elevation in each region).

The relationship between individual biomass and elevation was fitted using both linear and quadratic polynomial regression models, and model performance was compared using *F*-tests. The 95% confidence interval of the peak value was estimated using bootstrap resampling (Bootstrap, *R* = 1000). Analysis of covariance (ANCOVA) was used to test the net effect of elevation after controlling for mean DBH, with partial η^2^ used as the effect size measure. The allometric relationship between DBH and biomass was fitted using the power function equation *M* = *a*·*D^b^*. After logarithmic transformation, differences in the *b* value among elevations were compared, and the significance of slope differences was tested through the interaction between elevation and log (DBH). Differences in age structure were analyzed using the chi-square test. The growth process of DBH with age was fitted using generalized additive models (GAMs). The significance level was set at α = 0.05. Figures were produced using the ggplot2 package (Wickham, H.; Springer: New York, NY, USA, 2016).

## 3. Results and Analysis

### 3.1. Effects of Elevation on Biomass in Moso Bamboo Forests

**Figure 1:**
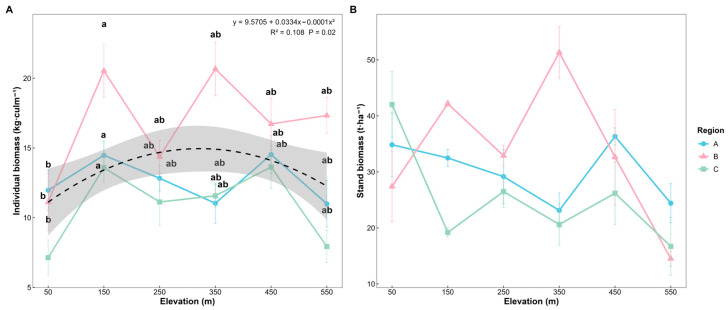
Changes in individual biomass and stand biomass of Moso bamboo at different elevations. (**A**)Individual biomass trends by region; (**B**) Stand biomass trends by region. Solid lines represent individual biomass (left axis) and dashed lines represent stand biomass (right axis). The dashed line in panel B represents the overall quadratic regression fit. Different colors represent different regions (A, B, C). Error bars indicate standard error (*n* = 3). Different superscript letters (a, b) indicate significant differences among elevations at *p* < 0.05 based on Tukey’s HSD test. The quadratic regression equation, R^2^, and *p*-value are shown in the upper right corner.

Two-way ANOVA showed that elevation had a highly significant effect on individual biomass (*p* < 0.001). The main effect of region was also highly significant (*p* < 0.001), and the region × elevation interaction was significant (*p* = 0.048). Individual biomass exhibited a clear unimodal pattern with increasing elevation (Figure 1). It was only 10.07 ± 3.31 kg culm^−1^ at 50 m, increased to a peak of 16.20 ± 3.88 kg culm^−1^ at 150 m, and was therefore 60.9% higher than that at 50 m (Table 2). Tukey’s HSD multiple comparisons indicated that individual biomass at 150 m was significantly higher than that at 50 m (*p* < 0.05). Thereafter, individual biomass fluctuated downward, declining to 12.08 ± 4.64 kg culm^−1^ at 550 m, representing a 25.4% decrease relative to the peak value. The quadratic regression model performed significantly better than the linear model (*p* < 0.05). Stand biomass was also significantly affected by elevation, region, and their interaction, all at highly significant levels (*p* < 0.001). Stand biomass reached its maximum at 50 m (34.75 ± 10.97 t·ha^−1^), decreased to 31.28 ± 10.11 t·ha^−1^ at 150 m, and further declined to 18.54 ± 7.13 t·ha^−1^ at 550 m, representing a 46.7% reduction compared with that at 50 m (Table 2). Unlike the unimodal elevational pattern of individual biomass, stand biomass showed an overall decline with increasing elevation, indicating a clear decoupling between the two elevational patterns.

Analysis of covariance further clarified the mechanisms underlying elevational variation in biomass (Table 3). After controlling for mean DBH, the effect of elevation on individual biomass changed from highly significant (*p* < 0.001) to non-significant at the α = 0.05 level (*p* = 0.051), and partial η^2^ declined from 0.48 to 0.21, corresponding to a 56% reduction. This result indicates that the effect of elevation on individual biomass was substantially weakened after accounting for DBH, suggesting that DBH captures much of the variation in individual biomass that would otherwise be attributed to elevation.

Validation using linear mixed-effects models: To account for potential spatial non-independence among the three plots within each study site, we fitted linear mixed-effects models (LMMs) with “site” as a random intercept. The fixed effects included elevation and region. The LMM results (Tables S1–S4) confirmed the main findings: elevation had a significant effect on individual biomass (*F* = 6.90, *p* = 0.002), and region also showed a significant effect (*F* = 15.04, *p* < 0.001), as determined by Satterthwaite’s approximation. The random-effect variance component for “site” was 11.50 (SD = 3.39), accounting for a substantial portion of the total variance, while the residual variance was negligible (5.37 × 10^−6^). Importantly, when site means were used as the experimental unit in a conservative analysis, the unimodal trend of individual biomass along elevation persisted, although the statistical significance was reduced due to the smaller sample size (*F* = 1.12, *p* = 0.399; Table S5). These results confirm that the main conclusions are robust to the nested sampling design.

### 3.2. Effects of Elevation on Biomass Allocation Patterns and Stand Structure

**Figure 2:**
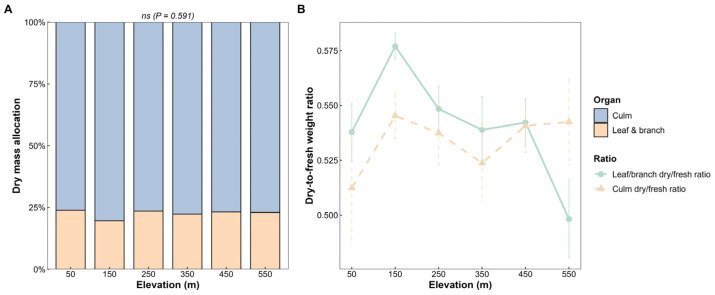
Biomass allocation and the dry-to-fresh weight ratio of Moso bamboo at different elevations. (**A**) Dry mass allocation proportions of leaf and branch and culm. (**B**) Dry-to-fresh weight ratios of leaf and branch and culm. Error bars indicate standard error (*n* = 9). “ns” in panel A indicates no significant difference among elevations *(p* = 0.591, Tukey’s HSD test).

The aboveground biomass allocation pattern of Moso bamboo along the elevational gradient showed a tendency toward greater culm allocation at intermediate elevations and greater branch and leaf allocation at both lower and higher elevations, although these differences were not statistically significant (*p* = 0.591) (Figure 2A). The proportion of branch and leaf biomass was 23.9% ± 6.0% at 50 m, declined to its lowest value of 19.7% ± 5.0% at 150 m, and then remained relatively stable between 22.4% and 23.5% from 250 to 550 m. Culm dry mass proportion showed the opposite trend, reaching a maximum of 80.3% ± 5.0% at 150 m (Table 2). Differences in the allocation proportion of branches and leaves among elevations were not statistically significant (*p* = 0.591), indicating that Moso bamboo maintained a relatively stable aboveground allocation pattern across elevations. The dry-to-fresh mass ratio of branches and leaves exhibited a distinct peak at 150 m, reaching 0.70 ± 0.02, which was 41.5% higher than that at 50 m, whereas values at the other elevations remained stable between 0.50 and 0.55 (Figure 2B, Table 2), suggesting lower water content in branches and leaves at 150 m.

**Figure 3:**
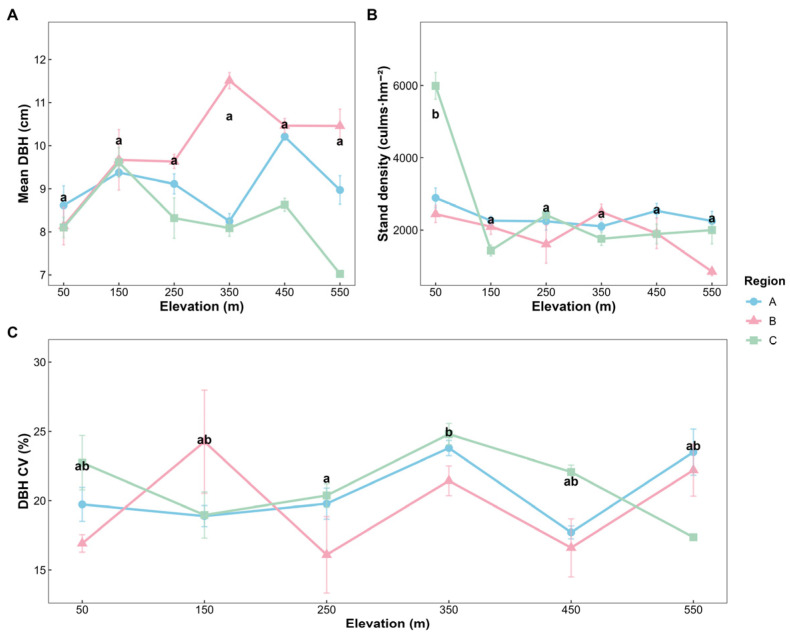
Changes in stand structure indices of Moso bamboo forests at different elevations. (**A**) Mean DBH; (**B**) stand density; and (**C**) coefficient of variation of DBH. Different colors represent different regions (A, B, and C). Different superscript letters indicate significant differences among elevations at *p* < 0.05 based on Tukey’s HSD test. Error bars indicate standard error (*n* = 3).

Stand density declined with increasing elevation (Figure 3B), from a maximum of 3775 ± 1733 culms ha^−1^ at 50 m to 1700 ± 769 culms ha^−1^ at 550 m, representing a decline of 55%. Mean DBH initially increased and then decreased along the elevational gradient (Figure 3A), reaching its maximum value of 9.77 ± 0.88 cm at 450 m. Elevation had strong effects on both mean DBH and stand density, with both effects being highly significant (*p* < 0.001). The coefficient of variation in DBH was lowest at 450 m (18.79%), indicating that stand structure was most uniform at this elevation.

### 3.3. Effects of Elevation on the Allometric Relationship Between DBH and Biomass

**Figure 4:**
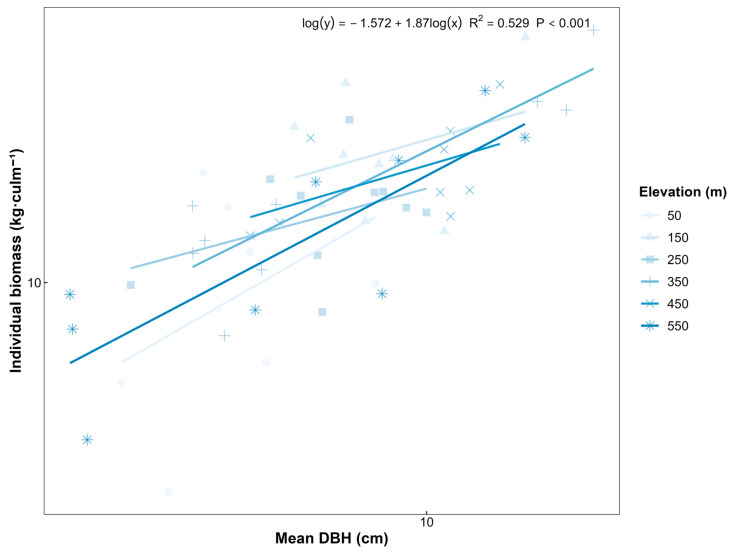
Allometric relationship between DBH and individual biomass of Moso bamboo.

The allometric equation showed a highly significant linear relationship in double-logarithmic coordinates (Figure 4, *p* < 0.001), indicating that DBH is a reliable predictor of individual biomass. Elevation-specific fitting showed that the b values at 350 m (*b* = 1.74, *p* = 0.001) and 550 m (*b* = 1.85, *p* = 0.005) were significantly greater than 1, indicating a tendency toward positive allometric growth. By contrast, the *b* values at 50, 150, 250, and 450 m were not significant (*p* = 0.135–0.411). ANCOVA of the allometric relationship showed that the interaction between elevation and log (DBH) was not significant (*F* = 0.35, *p* = 0.882), indicating that allometric slopes did not differ significantly among elevations. This suggests that the DBH–biomass allometric relationship of Moso bamboo remained relatively stable across elevations. In other words, elevation was associated with individual biomass mainly through its association with individual DBH (a size effect), rather than by altering the allometric relationship itself (a proportional effect). This finding of relative allometric stability is consistent with the mediating effect of DBH revealed by the ANCOVA and together provides a strong explanatory framework for the decoupling between individual and stand-level biomass patterns.

**Figure 5:**
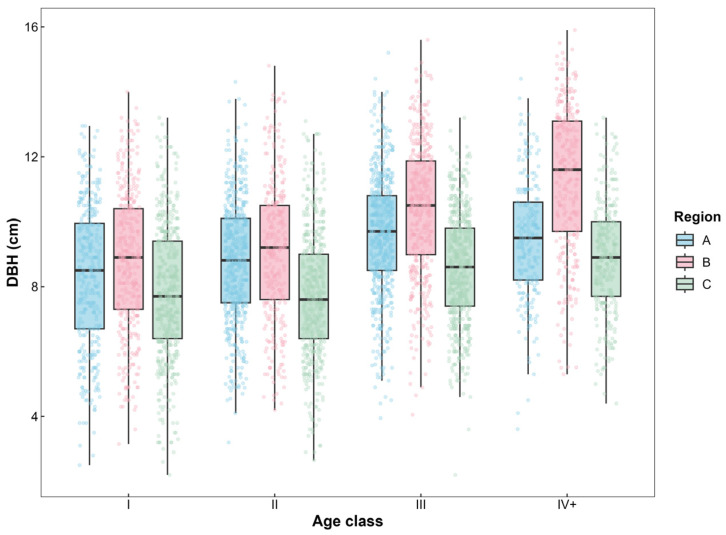
Boxplots of culm diameter at breast height (DBH) of Moso bamboo (*Phyllostachys edulis*) across different age classes in three regions.

Figure 5 shows that Region C generally had higher median DBH values across most age classes, whereas Region A tended to have lower values. However, substantial individual variation was observed within each age class, particularly among culms in Degree IV and above, where the dispersion was greatest. Individual culm size appears to depend more strongly on site conditions and the intensity of clonal integration in the year of shoot emergence than on culm age itself. Therefore, differences in individual biomass along the elevational gradient were achieved through changes in the size of newly emerged shoots in a given year, that is, through DBH, rather than through age-related accumulation of secondary growth.

## 4. Discussion

### 4.1. Overall Biomass Responses of Moso Bamboo to Elevation and the Mechanism Underlying Individual Stand Decoupling

In this study, the individual biomass of Moso bamboo showed a unimodal response to increasing elevation, peaking at approximately 150 m and being significantly higher than at 50 m. This result is consistent with findings from other mountain ecosystems, where plant productivity often peaks at mid-elevations due to optimal combinations of temperature, moisture, and nutrient availability [4,8]. For bamboo species specifically, similar mid-elevation optima have been reported for other clonal bamboos in the Himalayas and East Asia [25,26]. The lowest individual biomass occurred at low elevation (50 m), which can be mainly attributed to the excessively high stand density (3775 culms ha^−1^) and the resulting intense intraspecific competition, in accordance with the self-thinning rule [27]. At high elevation (550 m), individual biomass declined by 25.4% relative to the peak value, consistent with observations that bamboo yield decreases with increasing elevation [28]. The significant region × elevation interaction (*p* = 0.048) indicates that the position of the optimal elevation varies among regions, which differs from conclusions drawn from single-region studies and highlights the value of the multi-region design adopted here. The unimodal pattern of individual biomass along elevation is also consistent with the predictions of the mid-domain effect, which posits that geometric constraints may cause peak abundance or biomass at the center of a bounded spatial domain [6,7]. However, our data alone cannot distinguish between geometric and environmental drivers. We speculate that the observed peak at 150 m may reflect the combined influence of geometric constraints and environmental optima (e.g., temperature–moisture balance), but this hypothesis requires further testing with environmental measurements across a broader elevational range.

A clear decoupling relationship between individual biomass and stand biomass was observed in Moso bamboo forests in central-southern Jiangxi. Individual biomass peaked at 150 m, whereas stand biomass was highest at 50 m. This decoupling resulted from asynchronous elevational trends in the two components of stand biomass: stand density declined continuously from low to high elevation (a decrease of 55%), whereas individual biomass followed a unimodal curve. The product of these two components produced a trade-off pattern in which high stand biomass at low elevation was maintained by high density, but at the expense of individual growth. Similar trade-offs between density and individual size have been reported in other clonal plants and tree populations along stress gradients [29,30]. The ANCOVA results controlling for the elevational effect on DBH confirmed the substantial role of DBH in accounting for elevation-related variation and support the conclusion that DBH is the primary factor through which elevation is associated with individual biomass, although elevation may also exert weak effects through secondary pathways, such as height-to-diameter ratio or wood density. In this study, the conversion relationship between DBH and biomass remained relatively stable across elevations. Thus, elevation primarily affected individual biomass through a “size effect” (by changing DBH), rather than a “proportional effect” (by altering allocation strategy or the allometric relationship itself). This logical pathway, namely “elevation→DBH→individual biomass”, provides a strong explanatory framework for the decoupling between individual and stand-level biomass.

Globally, biomass estimation at regional scales must account for the integrated effects of multiple factors, and developing reliable biomass estimation models is a central task for quantifying carbon sequestration in forest ecosystems [31,32]. Climate-sensitive mixed-effects models provide a methodological framework for integrating multiple environmental variables into biomass estimation [33]. In the present study, linear mixed-effects models confirmed a significant regional effect, and the conservative analysis based on site means showed the same numerical trends in individual biomass, indicating that the main conclusions were only weakly affected by the choice of statistical method. Therefore, when constructing large-scale biomass estimation models for Moso bamboo, DBH can be used as the core predictor, whereas the effect of elevation may be incorporated mainly through its influence on DBH. Future studies could build mixed-effects biomass models for Moso bamboo by taking the DBH–biomass relationship as the core structure and introducing regional and climatic random effects, thereby improving both predictive accuracy and environmental adaptability.

It is important to note that the elevation of maximum mean DBH (450 m, 9.77 cm) did not coincide with the elevation of maximum individual biomass (150 m, 16.20 kg·culm^−1^). This apparent discrepancy arises because individual biomass is a function of both DBH and the allometric relationship between DBH and biomass, as well as stand density. At 450 m, although the mean DBH was the largest, the relatively lower density and potentially different allocation patterns resulted in lower individual biomass compared to 150 m. This underscores the importance of considering multiple size-related variables—not DBH alone—when evaluating bamboo productivity. For management purposes, individual biomass (which integrates DBH and density effects) is a more relevant indicator of productivity than DBH alone.

### 4.2. Conservatism in Biomass Allocation Patterns and Differences in Stand Structure

The aboveground biomass allocation pattern of Moso bamboo showed a tendency toward higher branch-and-leaf proportions at both low and high elevations and lower proportions at intermediate elevations, although these differences were not statistically significant (*p* = 0.591). Previous harvest-based studies in other bamboo species have reported similar allocation plasticity [34,35]. In Moso bamboo, culm biomass accounts for approximately 75% of total aboveground biomass at the individual level, representing the overall average allocation pattern [36]. In the present study, the culm proportion at 150 m (80.3%) exceeded this overall mean, possibly because favorable site conditions at low to mid elevations promote greater biomass investment in culms [37], as suitable hydrothermal conditions and soil nutrient availability facilitate the allocation of more photosynthates to supporting structures. Studies on other clonal plants, such as *Artemisia* species along elevational gradients, have shown both plastic and conserved allocation strategies depending on genetic constraints [18]. In contrast, a study of five typical bamboo species at different elevations in the Wuyi Mountains found that these species generally allocated more biomass to leaves at low elevations and more to stems at high elevations [38]. This pattern is not fully consistent with our finding that culm allocation in Moso bamboo was highest at 150 m. In addition to differences in study scale, this discrepancy may reflect fundamental differences in clonal architecture between Moso bamboo (a monopodial running bamboo) and the sympodial clumping bamboos examined in that study. Running bamboos rely on underground rhizomes for clonal propagation and resource integration, and their aboveground biomass allocation strategies may therefore differ substantially from those of clumping bamboos. In the present study, elevation did not significantly affect the proportion of branches and leaves (*p* = 0.591), indicating strong genetic conservatism, which contrasts with the plastic allocation strategy reported in some annual plants [18]. The branch-and-leaf dry-to-fresh mass ratio reached its highest value at 150 m (0.75), suggesting that approximately 150 m may represent the optimal elevation for Moso bamboo in central-southern Jiangxi, where photosynthetic activity is more vigorous and carbon assimilation products accumulate more fully, thereby promoting the synthesis of structural compounds. The exact mechanism remains to be verified in future studies through combined measurements of leaf functional traits, such as specific leaf mass and leaf nitrogen content, and non-structural carbohydrates. The culm dry-to-fresh mass ratio remained highly stable, with a coefficient of variation of only about 3%, indicating strong genetic conservatism in culm material properties, which is of practical importance for bamboo processing and utilization [39,40]. Taken together, both the allocation pattern (branch-and-leaf/culm ratio) and the allometric relationship (*b* value) were relatively conserved, and neither was significantly affected by elevation (*p* = 0.591; *p* = 0.882). This finding supports a complete mechanistic chain in which size effects dominate, whereas proportional effects remain weak.

In this study, stand density declined continuously with increasing elevation, in full agreement with the elevational pattern reported for bamboo forests at the southern margin of the Moso bamboo distribution range [41]. Mean DBH, however, first increased and then declined, which differs from a previous observation of a continuous decrease in mean DBH [41]. This discrepancy may be related to the narrower elevational range in the earlier study, which did not exceed 450 m. Elevation had highly significant effects on both stand density and mean DBH, further supporting the general pattern that Moso bamboo populations adapt to environmental gradients through a trade-off between density and individual size. Sparse planting tends to increase differences in individual biomass, whereas dense planting suppresses individual growth [42].

Our findings suggest that low-density areas at approximately 150 m in central-southern Jiangxi, with stand densities around 2000 culms·ha^−1^, may be promising for cultivating large-diameter Moso bamboo. However, this recommendation should be validated through targeted management trials before operational application. For high-density stands at 50 m (>3500 culms·ha^−1^), thinning to reduce stand density to approximately 2500–3000 culms·ha^−1^ may alleviate competition, but this should be tested experimentally. For areas above 450 m, we hypothesize that a stronger emphasis on ecological conservation rather than timber production may be appropriate, but this suggestion requires further validation under different environmental conditions.

### 4.3. Study Limitations and Future Directions

Several limitations of this study should be acknowledged. First, environmental variables such as temperature, soil moisture, and nutrient availability were not directly measured. Therefore, the mechanistic interpretations of elevational patterns, particularly those invoking hydrothermal conditions or soil fertility, remain speculative and should be tested in future studies that integrate direct environmental measurements with bamboo biomass data.

Second, this study was conducted within a single county (Wan’an County) in Jiangxi Province; the generalizability of our findings to other regions, particularly those with different climatic and edaphic conditions, requires further investigation.

Third, detailed management history (e.g., thinning, fertilization, and bamboo shoot harvesting) was not available for all plots, which may have influenced stand structure and productivity. Although we selected stands with no recorded major disturbances in the preceding five years, unrecorded historical management could still affect current biomass patterns.

Fourth, the nested sampling design introduces potential spatial pseudo-replication, although we used LMMs to statistically control for this issue. Future studies should consider increasing the number of independent sites per elevation and incorporating direct environmental measurements to achieve true replication and mechanistic understanding.

## 5. Conclusions

The individual biomass of Moso bamboo in central-southern Jiangxi exhibited a unimodal pattern with increasing elevation, reaching a peak at approximately 150 m, which appears to represent the optimal elevational zone for Moso bamboo growth. A clear decoupling relationship was observed between the elevational patterns of stand biomass and individual biomass, indicating that elevation induced a trade-off between stand density and individual size. Elevation was associated with individual biomass mainly through its association with DBH (a size effect), rather than by changing allocation strategy or the allometric relationship (a proportional effect), and this appears to be the primary factor underlying the decoupling between individual and stand-level biomass.

Our findings suggest that elevations around 150 m may be promising for large-diameter bamboo cultivation, although this recommendation should be validated through targeted management trials. In high-density stands at 50 m elevation, thinning to reduce stand density to approximately 2500–3000 culms·ha^−1^ may alleviate competition, but should be tested experimentally.For areas above 450 m, ecological conservation is suggested as a management hypothesis requiring further validation under varying environmental conditions.

## Figures and Tables

**Table 1:** Basic characteristics of the three study regions.

Region	Township	Coordinates	Parent Material	Soil Type	Soil Depth (cm)	Slope Gradient (°)
A	Wufeng	114°45′ E, 26°30′ N	Granite	Red soil	30–60	15–25
B	Gaobei	114°50′ E, 26°35′ N	Sandstone-shale	Yellow-red soil	20–60	20–30
C	Jiantou	114°42′ E, 26°28′ N	Granite	Red soil	35–55	10–20

**Table 2:** Biomass allocation and stand structure characteristics of Moso bamboo at different elevations (mean ± SD, *n* = 9).

Elevation (m)	Individual Biomass (kg·culm^−1^)	Stand Biomass (t·ha^−1^)	Leaf + Branch Proportion (%)	Culm Proportion (%)	Leaf + Branch DW/FW Ratio	Culm DW/FW Ratio	Mean DBH (cm)	Stand Density (culms·ha^−1^)	DBH CV (%)
50	10.07 ± 3.31 ^b^	34.75 ± 10.97	23.9 ± 6.0 ^a^	76.1 ± 6.0	0.53 ± 0.04	0.52 ± 0.08	8.28 ± 0.63 ^a^	3775 ± 1733 ^b^	19.80 ± 3.27 ^ab^
150	16.20 ± 3.88 ^a^	31.28 ± 10.11	19.7 ± 5.0 ^a^	80.3 ± 5.0	0.75 ± 0.02	0.55 ± 0.03	9.55 ± 0.70 ^a^	1928 ± 451 ^a^	20.71 ± 4.48 ^ab^
250	12.93 ± 2.51 ^ab^	29.85 ± 5.78	23.5 ± 4.4 ^a^	76.5 ± 4.4	0.55 ± 0.03	0.53 ± 0.04	9.08 ± 0.74 ^a^	2040 ± 754 ^a^	18.49 ± 3.97 ^a^
350	14.42 ± 5.19 ^ab^	31.69 ± 15.85	22.4 ± 8.4 ^a^	77.6 ± 8.4	0.54 ± 0.05	0.52 ± 0.05	9.28 ± 1.70 ^a^	2119 ± 417 ^a^	23.35 ± 1.95 ^b^
450	14.96 ± 2.77 ^ab^	31.72 ± 9.95	23.2 ± 4.7 ^a^	76.8 ± 4.7	0.55 ± 0.03	0.54 ± 0.04	9.77 ± 0.88 ^a^	2111 ± 567 ^a^	18.79 ± 3.15 ^ab^
550	12.08 ± 4.64 ^ab^	18.54 ± 7.13	23.0 ± 6.4 ^a^	77.0 ± 6.4	0.50 ± 0.05	0.54 ± 0.06	8.82 ± 1.56 ^a^	1700 ± 769 ^a^	21.03 ± 3.55 ^ab^

**Note:** Different superscript letters within the same column indicate significant differences at *p* < 0.05 based on Tukey’s HSD post hoc test. DWFW: fresh weight/dry weight ratio; DBH: diameter at breast height; CV: coefficient of variation.

**Table 3:** Results of analysis of covariance (ANCOVA) for individual culm biomass.

Source of Variation	df	Sum Sq	Mean Sq	F Value	*p* Value	Partial η^2^	95% CI for η^2^
Mean DBH (covariate)	1	517.106	517.106	80.435	<0.001 ***	0.636	[0.493, 1.000]
Region	2	42.113	21.056	3.275	0.047 *	0.125	[0.001, 1.000]
Elevation	5	77.320	15.464	2.405	0.051	0.207	[0.000, 1.000]
Residuals	46	295.726	6.429	—	—	—	—

**Note:** *** *p* < 0.001; * *p* < 0.05. Partial η^2^ values are reported with 95% confidence intervals estimated via bootstrap. DBH: diameter at breast height.

## Data Availability

The datasets presented in this article are not readily available because the data are part of an ongoing long-term monitoring study and have not yet been fully published. Requests to access the datasets should be directed to the corresponding author, Guanglu Liu (liuguanglu@icbr.ac.cn).

## Supplementary Materials

The following supporting information can be downloaded at: https://www.mdpi.com/article/10.3390/plants15142190/s1, Table S1. Linear mixed-effects model (LMM) fixed effects for individual biomass. Table S2. Linear mixed-effects model (LMM) random effects. Table S3. Linear mixed-effects model (LMM) ANOVA (Satterthwaite). Table S4. Likelihood ratio test for elevation effect in LMM. Table S5. Conservative analysis (site means as experimental unit): ANOVA results for individual biomass. Table S6. Effect sizes (partial η^2^) for ANOVA and ANCOVA models.
