# Supplementary material for: Effects of Elevational Gradient on Biomass Allocation Patterns of Moso Bamboo Forests in Central-Southern Jiangxi, China
Source: Plants (Basel). 2026 Jul 17;15(14):2190. doi: 10.3390/plants15142190 (PMC13414979; doi:10.3390/plants15142190)
Supplement: Supplementary file 1 [file plants-15-02190-s001.zip › plants-4395946-supplementary.pdf]

Supplementary Materials

Manuscript Title: Effects of elevational gradient on biomass allocation patterns of Moso bamboo forests in central-southern Jiangxi, China

Manuscript ID: plants-4395946

Authors: Shan Li, Jialin Fan, Xiaotong Liu, Jiajun Liu, Zhoubin Huang, Jingyao Zhang, Guanglu Liu
Corresponding Author: Guanglu Liu (liuguanglu@icbr.ac.cn)

Contents

Table S1. Linear mixed-effects model (LMM) fixed effects for individual biomass.
Table S2. Linear mixed-effects model (LMM) random effects.
Table S3. Linear mixed-effects model (LMM) ANOVA (Satterthwaite).
Table S4. Likelihood ratio test for elevation effect in LMM.
Table S5. Conservative analysis (site means as experimental unit): ANOVA results for individual biomass.
Table S6. Effect sizes (partial η^2) for ANOVA and ANCOVA models.

Table S1. Linear mixed-effects model (LMM) fixed effects for individual biomass.

Fixed effect	Estimate	Std. Error	df	t value	*P* value	95% CI (lower)	95% CI (upper)
Intercept	12.636	0.799	51.00	15.811	**<0.001**	11.069	14.202
Elevation	7.35×10^{-8}	1.64×10^{-5}	51.00	0.004	0.996	-3.20×10^{-5}	3.22×10^{-5}
Region B	4.231	1.130	51.00	3.743	**<0.001**	2.015	6.446
Region C	-1.808	1.130	51.00	−1.600	0.116	−4.024	0.407

Note: LMM: linear mixed-effects model. Site was included as a random intercept. Fixed effects were estimated using restricted maximum likelihood (REML) with Satterthwaite's approximation for degrees of freedom. Region A served as the reference level for region effects. Significant P values (*P<0.05*) are shown in bold.

Table S2. Linear mixed-effects model (LMM) random effects.

Random effect	Variance	Std. Deviation
Site (intercept)	11.496	3.391
Residual	5.37×10^{-6}	0.002

Note: LMM: linear mixed-effects model. Site was included as a random intercept to account for spatial non-independence among plots within the same study site. The residual variance was negligible, indicating that most of the variation was captured at the site level.

Table S3. Linear mixed-effects model (LMM) ANOVA (Satterthwaite).

Effect	Sum Sq	Mean Sq	NumDF	DenDF	*F* value	*P* value
Elevation	1.08×10^{-10}	1.08×10^{-10}	1	51.00	2.01×10^{-5}	0.996
Region	1.61×10^{-4}	8.07×10^{-5}	2	51.00	15.041	**<0.001**

Note:LMM: linear mixed-effects model. Satterthwaite's approximation was used for denominator degrees of freedom. Significant *P* values (*P< .05*) are shown in bold.

Table S4. Likelihood ratio test for elevation effect in LMM.

Model	npar	AIC	BIC	logLik	Deviance	Chisq	Df	*P* value
Null model (region only)	5	267.89	277.93	-128.94	257.89	—	—	—
Full model (elevation+region)	6	269.27	281.31	-128.63	257.27	0.621	1	0.431

Note: LMM: linear mixed-effects model. Null model: `bio_single ~ region + (1 | site)`. Full model: `bio_single ~ elevation + region + (1 | site)`. The likelihood ratio test indicated that adding elevation as a fixed effect did not significantly improve model fit (*P=0.431*).

Table S5. Conservative analysis (site means as experimental unit): ANOVA results for individual biomass.

Source	df	Sum Sq	Mean Sq	F value	P value
Elevation	5	73.56	14.71	1.124	0.399
Residuals	12	157.07	13.09	—	—

Note: In this conservative analysis, the mean individual biomass of the three plots within each site was used as the experimental unit (n=3 per elevation per region). The unimodal trend of individual biomass along elevation persisted (peak at 150 m), although statistical significance was reduced due to the smaller effective sample size, confirming that the main conclusions are robust to the nested sampling design.

Table S6. Effect sizes (partial η^2) for ANOVA and ANCOVA models.

Model	Parameter	Partial η^2	95% CI (lower)	95% CI (upper)
Two-way ANOVA	Region	0.581	0.393	1.000
	Elevation	0.483	0.235	1.000
	Region×Elevation	0.364	0.003	1.000
ANCOVA	Mean DBH	0.636	0.493	1.000
	Region	0.125	0.001	1.000
	Elevation	0.207	0.000	1.000

Note: Partial η^2 values are reported with 95% confidence intervals estimated via bootstrap (R=1000). DBH: diameter at breast height. The large effect size for mean DBH (0.636) confirms its dominant role as a predictor of individual biomass.
